# Orthogonal Procrustes Analysis for Dictionary Learning in Sparse Linear Representation

**DOI:** 10.1371/journal.pone.0169663

**Published:** 2017-01-19

**Authors:** Giuliano Grossi, Raffaella Lanzarotti, Jianyi Lin

**Affiliations:** 1 Department of Computer Science, University of Milan, Via Comelico 39, 20135 Milan, Italy; 2 Department of Applied Mathematics and Sciences, Khalifa University, Al Saada St., PO Box 127788, Abu Dhabi, United Arab Emirates; National University of Defense Technology College of Mechatronic Engineering and Automation, CHINA

## Abstract

In the sparse representation model, the design of overcomplete dictionaries plays a key role for the effectiveness and applicability in different domains. Recent research has produced several dictionary learning approaches, being proven that dictionaries learnt by data examples significantly outperform structured ones, e.g. wavelet transforms. In this context, learning consists in adapting the dictionary atoms to a set of training signals in order to promote a sparse representation that minimizes the reconstruction error. Finding the best fitting dictionary remains a very difficult task, leaving the question still open. A well-established heuristic method for tackling this problem is an iterative alternating scheme, adopted for instance in the well-known K-SVD algorithm. Essentially, it consists in repeating two stages; the former promotes sparse coding of the training set and the latter adapts the dictionary to reduce the error. In this paper we present R-SVD, a new method that, while maintaining the alternating scheme, adopts the Orthogonal Procrustes analysis to update the dictionary atoms suitably arranged into groups. Comparative experiments on synthetic data prove the effectiveness of R-SVD with respect to well known dictionary learning algorithms such as K-SVD, ILS-DLA and the online method OSDL. Moreover, experiments on natural data such as ECG compression, EEG sparse representation, and image modeling confirm R-SVD’s robustness and wide applicability.

## 1 Introduction

In many application domains, such as denoising, classification and compression of signals [1–3], it is often convenient to use a compact signal representation following Occam’s Razor principle. Dimensionality reduction can be accomplished either with feature selection [4, 5] or sparse decomposition techniques [6].

Sparsity is a classical linear algebra approach leading to parsimonious representation. Consider an overcomplete *dictionary* matrix $D\in R^{n\times m}$ (*n* < *m*) with columns *d*_*i*_, *i* = 1,…,*m*, called atoms, and a signal vector $y\in R^{n}$; the sparsity approach consists in expressing *y* as linear combination ∑_*i*_ *x*_*i*_*d*_*i*_ with as few as possible non-zero coefficients $x_{i}\in R$. Formally, the *sparse approximation* problem consists in finding $x\in R^{m}$ minimizing the least squares error ‖*y* − *Dx*‖_2_ under the constraint that its *ℓ*_0_-norm ‖*x*‖_0_ ≔ #{*i*: *x*_*i*_ ≠ 0} be at most a threshold k∈N, i.e. *x* is *k* *-sparse*. This problem is combinatorial in nature and hence NP-hard [7]. Specifically, the sparse representation of a given set of probe signals poses two relevant questions.

The first concerns the development of efficient algorithms for solving the sparse approximation problem. To mention just a few, we recall those based on *ℓ*_0_-minimization such as the greedy method Orthogonal Matching Pursuit (OMP) [8], the iterative methods *k*-Limaps (Lipschitizian Mappings for Sparsity) [9], and SL0 (Smoothed L0) [10], or those based on *ℓ*_1_-minimization such as Basis Pursuit (BP) [11] and the Lasso [12].

The second issue, that we tackle in this article, concerns the design of suitable dictionaries that adaptively capture the model underlying the data. In literature, the proposed methods of *dictionary design* can be classified into two types [6].

The former consists in building *structured dictionaries* generated from analytic prototype signals. For instance, these comprise dictionaries formed by set of time-frequency atoms such as window Fourier frames and Wavelet frames [13], adaptive dictionaries based on DCT [14], Gabor functions [15], bandelets [16] and shearlets [17].

The latter type of design methods arises from the machine learning field and consists in *training a dictionary* from available signal examples, that turns out to be more adaptive and flexible for the considered data and task. The first approach in this sense [18] proposes a statistical model for natural image patches and searches for an overcomplete set of basis functions (dictionary atoms) maximizing the average log-likelihood (ML) of the model that best accounts for the images in terms of sparse, statistically independent components. In [19], instead of using the approximate ML estimate, a dictionary learning algorithm is developed for obtaining a Bayesian MAP-like estimate of the dictionary under Frobenius norm constraints. The use of Generalized Lloyd Algorithm for VQ codebook design suggested the iterative algorithm named MOD (Method of Optimal Directions) [20]. It adopts the alternating scheme, first proposed in [21], consisting in iterating two steps: signal sparse decomposition and dictionary update. In particular, MOD carries out the second step by adding a matrix of vector-directions to the actual dictionary.

Alternatively to MOD, the methods that use least-squares solutions yield optimal dictionary updating, in terms of residual error minimization. For instance, such an optimization step is carried out either iteratively in ILS-DLA [22] on the whole training set (i.e. as batch), or recursively in RLS-LDA [23] on each training vector (i.e. continuously). In the latter method the residual error includes an exponential factor parameter for forgetting old training examples. With a different approach, K-SVD [2] updates the dictionary atom-by-atom while re-encoding the sparse non-null coefficients. This is accomplished through rank-1 singular value decomposition of the residual submatrix, accounting for all examples using the atom under consideration. Recently, Sulam et al. [24] introduced OSDL, an hybrid version of dictionary design, which builds dictionaries, fast to apply, by imposing a structure based on a multiplication of two matrices, one of which is fully-separable cropped Wavelets and the other is sparse, bringing to a double-sparsity format.

In this work we propose R-SVD (Rotate-SVD), an algorithm for dictionary learning in the sparsity model, inspired by a type of statistical shape analysis, called Procrustes method [25] (named after the ancient Greek myth of Damastes, known as Procrustes, the “stretcher”, son of Poseidon, who used to offer hospitality to the victims of his brigandage compelling them to fit into an iron bed by stretching or cutting off their legs), which has applications also in other fields such as psychometrics [26] and crystallography [27]. In fact, it consists in applying Euclidean transformations to a set of vectors (atoms in our case) to yield a new set with the goal of optimizing the model fitting measure. While maintaining the alternating scheme, R-SVD algorithm splits the dictionary into several groups of atoms and applies the Orthogonal Procrustes analysis simultaneously to all the atoms in each group capturing more complex data structures and being more efficient. The technique is able to find an optimal dictionary after few iterations of the scheme. Notice that the proposed method differs from K-SVD [28], which instead updates one atom at a time together with the corresponding sparse coefficients. Several experimental sessions show that R-SVD is effective and behaves better than several well known dictionary learning algorithms such as K-SVD, ILS-DLA and the online method OSDL.

In Sec. 2 we describe the problem and the proposed R-SVD algorithm. In Sec. 3 we conduct an experimental analysis studying the group size parameter of the method (sec. 3.1), showing results on synthetic data (sec. 3.2), investigating the role of the sparse decomposition method (sec. 3.3), and comparing with other dictionary learning algorithms (sec. 3.4). In Sec. 4 we report applications to ECG signal compression (sec. 4.1), EEG signal representation (sec. 4.2), and image modeling (sec. 4.3). Finally, we draw some conclusions in Sec. 5.

## 2 Method

In this section we use the notation $A={\left\{a_{i}\right\}}_{i=1}^{q}\in R^{p\times q}$ to indicate a *p* × *q* real-valued matrix with columns $a_{i}\in R^{p},i=1,...,q$. Suppose we are given the training dataset $Y={\left\{y_{i}\right\}}_{i=1}^{L}\in R^{n\times L}$. The dictionary learning problem consists in finding an overcomplete dictionary matrix $D={\left\{d_{i}\right\}}_{i=1}^{m}\in R^{n\times m}$ (*n* < *m*), which minimizes the least squares errors $∥y_{i}-Dx_{i}{∥}_{2}^{2}$, so that all coefficient vectors $x_{i}\in R^{m}$ are *k*-sparse. Formally, by letting $X={\left\{x_{i}\right\}}_{i=1}^{L}\in R^{m\times L}$ denote the coefficient matrix, this problem can be precisely stated as

$$\underset{D\in R^{n\times m},X\in R^{m\times L}}{\mathrm{argmin}}{∥Y-DX∥}_{F}^{2}\;\text{subject}\;\text{to}\;{∥x_{i}∥}_{0}\leq k,\;i=1,...,L.$$ (1)

One can multiply the *i*-th column of *D* and divide the *i*-th row of *X* by a common non-null constant to obtain another solution attaining the same value. Hence, w.l.o.g. atoms in *D* are constrained to be unit *ℓ*_2_-norm, corresponding to vectors *d*_*i*_ on the unit (*n* − 1)-sphere $S^{n-1}$ centered at the origin.

The search for the optimal solution is a difficult task due both to the combinatorial nature of the problem and to the strong non-convexity given by the *ℓ*_0_ conditions. We tackle this problem adopting the well established alternating optimization scheme [21], which consists in repeatedly executing the two steps:

***Step 1*.** Sparse coding: solve problem (1) for *X* only (fixing the dictionary *D*)
***Step 2*.** Dictionary update: solve problem (1) for *D* only (fixing *X*).

In particular, for sparse decomposition in Step 1 we use the greedy algorithm OMP because of its simplicity yet efficiency. Clearly, other sparse recovery methods could be adopted (e.g. BP, Lasso, *k*-Limaps, SL0). Experimentally, we observe that this choice does not substantially affect R-SVD performances in comparison with K-SVD.

Step 2 represents the core of the R-SVD method as detailed in the following.

### 2.1 Dictionary learning by Procrustes analysis

Let us first recall the idea of the Procrustes analysis. It consists in applying affine transformations (e.g., moving, stretching and rotating) to a given geometrical object in order to best fit the shape of another one. When the admissible transformations are restricted to orthogonal ones, it is referred to as Orthogonal Procrustes analysis [25].

Basically, in the proposed method R-SVD, after splitting the dictionary *D* into atom groups, the Orthogonal Procrustes analysis is applied to each group to find the best rotation (either proper or improper) that minimizes the total least squares error. Consequently, each group is updated by the optimal affine transformation thus obtained. Formally, let us denote by [*m*] ≔ {1,…,*m*} the set of first *m* positive integers and let *I* ⊂ [*m*] denote a set of indices for matrix columns or rows. Given any index set *I* of size *s* = |*I*|, let $D_{I}\in R^{n\times s}$ be the submatrix (subdictionary) of *D* formed by the *columns* indexed by *I*, that is *D*_*I*_ = {*d*_*i*_}_*i* ∈ *I*_, and let $X_{I}\in R^{s\times L}$ be the submatrix of *X* formed by the *rows* indexed by *I*; hence *s* is the size of atom group *D*_*I*_. In this setting, we can decompose the product *DX* into the sum

$$DX=D_{I}X_{I}+D_{I^{c}}X_{I^{c}}$$

of a matrix *D*_*I*_*X*_*I*_ dependent on the group *I* and a matrix *D*_*I*^*c*^_ *X*_*I*^*c*^_ dependent on the complement *I*^*c*^ = [*m*] \ *I*. Therefore, the objective function in Eq (1) can be written as ${∥Y-DX∥}_{F}^{2}={∥Y-D_{I^{c}}X_{I^{c}}-D_{I}X_{I}∥}_{F}^{2}$.

Now, after isolating the term *D*_*I*_ *X*_*I*_ in ${∥Y-DX∥}_{F}^{2}$ and setting *E* ≔ *Y* − *D*_*I*^*c*^_*X*_*I*^*c*^_, one can consider the optimization problem

$$\underset{S\in R^{n\times s}}{\mathrm{argmin}}{∥E-SX_{I}∥}_{F}^{2}\;\;\text{subject}\;\text{to}\;\;S\subset S^{n-1}$$ (2)

that corresponds to solving a subproblem of Step 2 by restricting the update to group *D*_*I*_ of unit *ℓ*_2_-norm atoms.

Our method aims at yielding a new atom group $S=D_{I}^{\prime }$, in general suboptimal for problem (2), by an orthogonal transformation matrix *R* (i.e. *R*^*T*^ *R* = *I*) applied on *D*_*I*_, namely $D_{I}^{\prime }=RD_{I}$. The set of orthogonal matrices *R* of order *n*, called orthogonal group *O*(*n*) (not to be confused with group of atoms), can be partitioned into the special orthogonal subgroup *SO*(*n*) formed by proper rotations, i.e. those with det*R* = 1, and the set *O*(*n*) \ *SO*(*n*) of improper rotations (or rotoreflections), i.e. those with det*R* = −1. Therefore, the search for such an optimal transformation can be stated as the following minimization problem

$$\min_{R\in O(n)}\;{∥E-RH∥}_{F}^{2}$$ (3)

where $H≔D_{I}X_{I}\in R^{n\times L}$. Notice that in denoting *E* and *H* we omit the dependence on *I*. The problem (3) is known as the *Orthogonal Procrustes problem* [25] and can be interpreted as finding the rotation of a subspace matrix *H*^*T*^ to closely approximate a subspace matrix *E*^*T*^ [29, §12.4.1].

The orthogonal Procrustes problem admits (at least) one optimal solution $\hat{R}$ which is [29] the transposed orthogonal factor *Q*^*T*^ of the polar decomposition *EH*^*T*^ = *QP*, and can be effectively computed as $\hat{R}=Q^{T}=VU^{T}$ from the orthogonal matrices *U* and *V* of the singular value decomposition $EH^{T}=U\Sigma V^{T}\in R^{n\times n}$.

Hence the rotation matrix we seek is $\hat{R}=VU^{T}$, the new dictionary *D*′ has the old columns of *D* in the positions *I*^*c*^ and the new submatrix $D_{I}^{\prime }=\hat{R}D_{I}$ in the positions *I*, while the new non-increased value of reconstruction error is

$$∥Y-D^{\prime }{X∥}_{F}^{2}\;=\;∥Y-D_{I^{c}}X_{I^{c}}-VU^{T}D_{I}X_{I}{∥}_{F}^{2}\;\leq \;{∥Y-DX∥}_{F}^{2}.$$

At this point the idea of the whole algorithm is quite straight-forward:

1. at each dictionary update iteration (Step 2) partition the set of column indices [*m*] = *I*_1_ ⊔ *I*_2_ ⊔ … ⊔ *I*_*G*_ into *G* subsets,
2. then split *D* accordingly into atom groups *D*_*I*_*g*__, *g* = 1,…,*G*, and
3. update every atom group *D*_*I*_*g*__.

These updates can be carried out either in parallel or sequentially with some order. We have chosen the sequential update with ascending order of atom popularity, i.e. sorting the indices *i* ∈ [*m*] w.r.t. the usage of atom *d*_*i*_, computable as *ℓ*_0_-norm of the *i*-th row in *X*. For sake of simplicity we set uniformly the group size to *s* = |*I*_*g*_| for all *g*, possibly except the last group (*G* = ⌈*m*/*s*⌉) if *m* is not a multiple of *s*: |*I*_*G*_| = *m* − *Gs*. Regarding this choice, we have seen experimentally that our method is agnostic w.r.t. grouping criteria such as random balanced grouping, cumulative coherence based partitioning, and clustering by absolute cosine similarity.

After processing all *G* groups, the method moves to the next iteration, and goes on until a stop condition is reached (eg. the maximum number of iterations as commonly chosen, or an empirical convergence criterion based on successive iterates). The main steps can be summarized in Algorithm 1 (the Matlab code implementing the algorithm is available on the website http://phuselab.di.unimi.it/resources.php).

**Algorithm 1** R-SVD

**Input:** $Y\in R^{n\times L}$: column-vector signals for training the dictionary

**Output:** $D\in R^{n\times m}$: trained dictionary; $X\in R^{m\times L}$: sparse encoding of *Y*

1: Initialize dictionary *D* picking *m* examples from *Y* at random

2: **repeat**

3: Sparse coding: $X={\mathrm{argmin}}_{X}{∥Y-DX∥}_{F}^{2}$ subject to ‖*x*_*i*_‖_0_ ≤ *k* for *i* = 1,…,*L*

4: Partition indices [*m*] = *I*_1_ ⊔ *I*_2_ ⊔ … ⊔ *I*_*G*_ sorting by atom popularity

5: **for** *g* = 1,…,*G* **do**

6:  *J* = *I*_*g*_

7:  *E* = *Y* − *D*_*J*^*c*^_*X*_*J*^*c*^_

8:  *H* = *D*_*J*_*X*_*J*_

9:  $R={\mathrm{argmin}}_{R\in O(n)}{∥E-RH∥}_{F}^{2}=VU^{T}$ by rank-*s* SVD *EH*^*T*^ = *U*Σ*V*^*T*^

10:  *D*_*J*_ = *RD*_*J*_

11: **end for**

12: **return** *D*, *X*

13: **until** stop condition

Notice that in our method the renormalization of atoms to unit length at each iteration is not necessary since they are inherently yielded with such a property from this Procrustes analysis, and hence in practice some large part of renormalizing computations as in ILS-DLA [22] and K-SVD [28] can be avoided.

### 2.2 Computational time analysis

A useful computational speedup in the update of every group *D*_*I*_ can be described as follows. Let us pre-compute $XY^{T}\in R^{m\times n}$ and $XX^{T}\in R^{m\times m}$ at the beginning of each dictionary update (Step 2). The matrix *EH*^*T*^ undergoing the SVD can be computed as

$$EH^{T}=D_{I}\left[X_{I}Y^{T}-X_{I}{\left(X_{I^{c}}\right)}^{T}{\left(D_{I^{c}}\right)}^{T}\right]$$

where *D*_*I*_ and *D*_*I*^*c*^_ come from previous update step, and the term *X*_*I*_*Y*^*T*^ is the submatrix formed by rows *I* of *XY*^*T*^, while *X*_*I*_(*X*^*T*^)_*I*^*c*^_ by rows *I* and columns *I*^*c*^ of *XX*^*T*^. With elementary matrix products this computation requires *O*(*sn*(*n* + *m* − *s*)) flops, which is lower than *O*(*nmL*) since *s* < *n* < *m* ≪ *L*.

Notice that, since rank *HE*^*T*^ ≤ rank*H* ≤ *s*, it is not necessary to obtain the full SVD of *HE*^*T*^, but rather truncate the decomposition to the first *s* singular vectors *u*_*i*_, *v*_*i*_: $\hat{R}=VU^{T}=\sum_{i=1}^{s}v_{i}u_{i}^{T}$. We thus use the truncated SVD algorithm by [30] based on structured random matrix that requires *O*(*n*^2^ log *s*) flops. The computation of *HE*^*T*^ and its SVD is repeated *G* = ⌈*m*/*s*⌉ times. The computational time of R-SVD is dominated by the pre-computation of *XY*^*T*^ and *XX*^*T*^, and therefore taking into account the sparsity of matrix *X* the asymptotic estimate for one iteration of R-SVD is $T_{\text{R-SVD}}\left(k,n,m,L,s\right)=O\left(\left(\frac{k^{2}}{s}+\frac{k}{s}n\right)mL\right)$, compared to K-SVD’s iteration *T*_K-SVD_(*k*, *n*, *m*, *L*) = *O*((*k*^2^ + *n*)*mL*). Note that, when *s* = Ω(*k*) we have $\frac{k^{2}}{s}+\frac{k}{s}n=o\left(k+n\right)=o\left(k^{2}+n\right)$ implying that the computational time *T*_R-SVD_ is lower than *T*_K-SVD_.

## 3 Experimental analysis

In this section we test the proposed R-SVD algorithm devoting at first an in-depth analysis to how to choose the group size *s* defined above. Then we apply the method on synthetic data conducting extensive experiments on both R-SVD and K-SVD using OMP as sparsifier. A further investigation is conducted on two different sparse decomposition methods, namely *k*-Limaps and SL0, and alternative dictionary learning methods, namely ILS-DLA by Engan et al. [20] and OSDL by Sulam et al. [24].

Following [28], the dictionary $D\in R^{n\times m}$ is randomly drawn, with i.i.d. standard Gaussian distributed entries and each column normalized to unit *ℓ*_2_-norm. The training set $Y\in R^{n\times L}$ is generated column-wise by *L* linear combinations of *k* dictionary atoms selected at random, and by adding white Gaussian noise matrix *N* with various signal-to-noise ratio (SNR), i.e. *Y* = *DX* + *N*. We measure the performances of the algorithms in terms of the reconstruction error (or quality) expressed as ${\text{E}}_{\text{SNR}}=20\log_{10}{(∥Y∥}_{F}/∥Y-\tilde{D}\tilde{X}{∥}_{F})$ dB, where $\tilde{D}$ and $\tilde{X}$ are the learned dictionary and the sparse encoding matrix respectively.

### 3.1 Setting the group size

It is naturally expected that the group size *s* affects both reconstruction quality and running time. In order to give some insight on this parameter, we run R-SVD algorithm on synthetic training sets by setting *L* = 8000, $D\in R^{50\times 100}$, *k* = 5 and SNR = 30 dB for noise *N* and letting *s* range in the interval 1 ÷ 25. Notice that when *s* = 1, our method is similar to K-SVD [28] except in the recovery of the sparse coefficients yielded by SVD decomposition.

**Fig 1:**
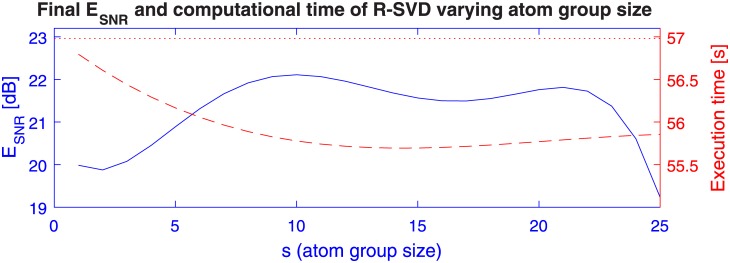
R-SVD’s dependency on the group size parameter *s*. Other experiment parameters are: training size *L* = 8000, dictionary size 50 × 100, additive noise of SNR = 30 dB, number of iterations *T* = 200. The lines (connecting points, for sake of readability) represent: average final E_SNR_ of the reconstructed dictionary (*solid blue curve*) w.r.t. the generating dictionary, computational time of the R-SVD (*dashed red curve*) and the K-SVD (*dotted red line*) in the dictionary learning task.

In Fig 1 we report the reconstruction error E_SNR_ (solid curve), and the computational times of both R-SVD (dashed curve) and K-SVD (dotted line), all averaged over 100 trials. It can be noticed that R-SVD method behaves better near the value *s* = 10. We thus choose this tradeoff setting for the experimental assessments of the method in the following sections.

### 3.2 Comparative results on synthetic data

In order to test the R-SVD method and compare it to the K-SVD algorithm, we first run experiments on random training instances. We consider dictionaries of size 50 × 100 and 100 × 200, dataset of size up to *L* = 10000 and sparsity *k* = {5, 10}. The algorithms K-SVD and R-SVD are run for *T* = 200 dictionary update iterations, that turns out to be sufficient to achieve empirical convergence of the performance measure. For each experimental setting we report the average error over 100 trials.

**Fig 2:**
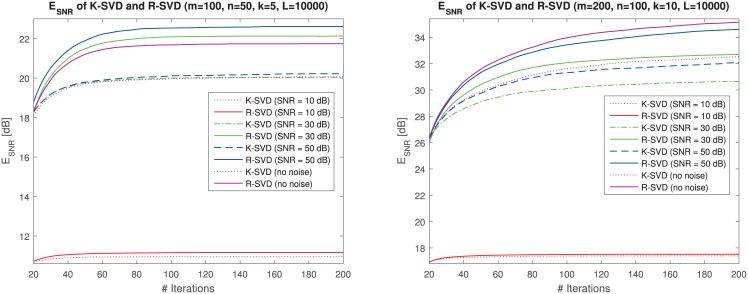
Average reconstruction error E_SNR_ in sparse representation using dictionary learnt by K-SVD (non-solid lines) and R-SVD (solid lines), for *L* = 10000 synthetic vectors varying the additive noise power (in the legend). Averages are calculated over 100 trials and plotted versus update iteration count. *Left*: $D\in R^{50\times 100}$ with sparsity *k* = 5, *Right*: $D\in R^{100\times 200}$ with sparsity *k* = 10.

In Fig 2 we highlight the learning trends of the two methods, plotting at each iteration count the E_SNR_ values on synthetic vectors *Y* = *DX* + *N*, varying the additive noise SNR = 10, 30, 50, ∞ (no noise) dB. It can be seen that, after an initial transient, the gap between R-SVD and K-SVD increases with the iteration count, establishing a final gap of 2 dB or more in conditions of middle-low noise power (SNR ≥ 30 dB).

**Fig 3:**
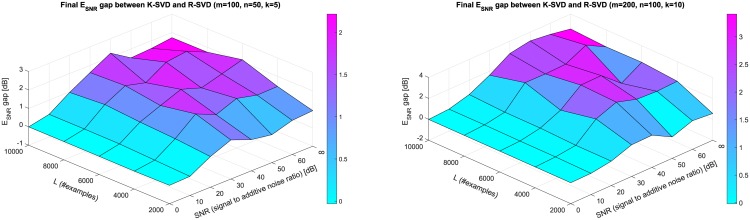
Gap between final (*T* = 200) E_SNR_ of K-SVD and R-SVD obtained with all parameter combinations *L* = 2000, 4000, 6000, 8000, 10000 and SNR = 0, 10, 20, 30, 40, 50, 60, ∞ (no noise). Results are averages over 100 trials; points are interpolated with coloured piece-wise planar surface for sake of readability. *Left*: $D\in R^{50\times 100}$ with sparsity *k* = 5. *Right*: $D\in R^{100\times 200}$ with sparsity *k* = 10.

In order to explore the behavior of R-SVD and K-SVD in a fairly wide range of parameter values, we report in Fig 3 the gaps between their final (*T* = 200) reconstruction error E_SNR_, varying *L* in 2000 ÷ 10000, noise SNR in 0 ÷ 60 dB, and in case of no noise. Dictionary sizes, sparsity and number of trials are set as above. When the additive noise power is very high (eg. SNR = 0 or 10 dB) the two methods are practically comparable, probably because the presence of significant noise would mislead any learning algorithm. On the other hand, when the noise is quite low the R-SVD algorithm outperforms K-SVD with a gap up to 3 dB.

Moreover, it is useful to evaluate the number of correctly identified atoms in order to measure the ability of the learning algorithms in recovering the original dictionary *D* from the noise-affected data *Y*. This is accomplished by maximizing the matching between atoms *d*_*i*_ of the original dictionary with atoms ${\tilde{d}}_{j}$ of the dictionary $\tilde{D}$ yielded by the algorithm: two atoms $\left(d_{i},{\tilde{d}}_{j}\right)$ are considered matched when their cosine distance is small [28], i.e. precisely

$$1-|d_{i}^{T}{\tilde{d}}_{j}|<\delta ≔0.01.$$

In Table 1 we report the average number of recovered atoms on random instances. Notice that R-SVD performs slightly better independently of the additive noise power.

**Table 1:** Average number of atoms correctly recovered (matched) by K-SVD and R-SVD algorithms at various SNR levels of additive noise on dictionary *D* of size 50 × 100 and 100 × 200. *L* = 10000, and remaining parameter values as in Fig 3.

Number of recovered atoms								
*n* × *m*	SNR = 10		SNR = 30		SNR = 50		no noise	
	K-SVD	R-SVD	K-SVD	R-SVD	K-SVD	R-SVD	K-SVD	R-SVD
50 × 100	94.52	97.37	92.15	94.08	92.1	93.84	92.07	94.03
100 × 200	195.82	199.02	192.42	194.98	192.49	194.57	192.87	194.7

### 3.3 Choice of the sparse decomposition method

So far we have used OMP as sparsifier for both R-SVD and K-SVD methods. Here we investigate R-SVD’s performances adopting the sparse decomposition techniques *k*-Limaps [9] and SL0 [10]. The former (developed by the authors) has proven its ability to perform better than other sparsity methods well know in literature (see experimental sections in [9]), while the latter is particularly suitable for fast applications.

**Fig 4:**
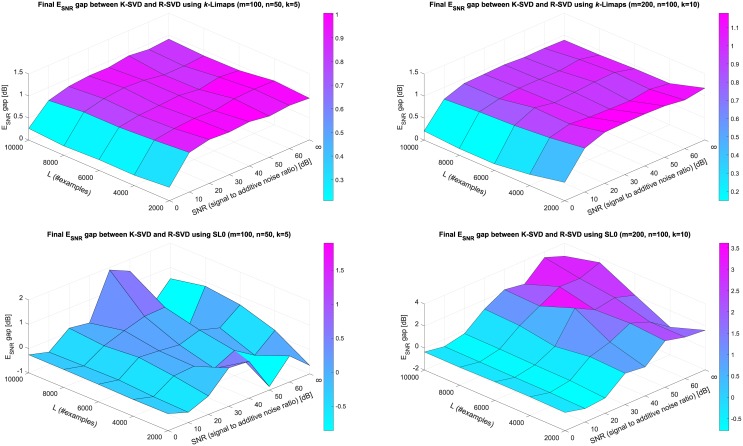
Comparison of K-SVD and R-SVD errors when *k*-Limaps (top) or SL0 (bottom) sparse decomposition methods are used. The surface represents the gap between final (*T* = 200) E_SNR_ of K-SVD and R-SVD obtained with all parameter combinations *L* = 2000, 4000, 6000, 8000, 10000 and SNR = 0, 10, 20, 30, 40, 50, 60, ∞ (no noise). Results are averages over 100 trials; points are interpolated with coloured piece-wise planar surface for sake of readability. *Left*: $D\in R^{50\times 100}$ with sparsity *k* = 5. *Right*: $D\in R^{100\times 200}$ with sparsity *k* = 10.

Each method has been applied to compute line 3 of Alg. 1, R-SVD, and the corresponding operation in the K-SVD algorithm. The SL0 (available on authors’ webpage http://ee.sharif.edu/~SLzero/) is set up with scale parameter *μ*_0_ = 2 and *σ* decrease factor equal to 4/5, while *k*-Limaps is initialized setting to 100 the maximum number of iterations. The experiments of R-SVD and K-SVD incorporating *k*-Limaps and SL0 were conducted under the same conditions of subsection 3.2. The resulting gap between final E_SNR_’s is shown in Fig 4.

Notice that, using *k*-Limaps as sparsifier, R-SVD provides performances that are almost uniformly and moderately better than K-SVD, unless the additive noise is very high. In such a case there is another evidence that the two algorithms are equally misled by noise-affected data. Moreover, while the behavior of the two algorithms incorporating SL0 is more contrasting, it can be seen however that R-SVD with SL0 usually performs better.

### 3.4 Alternative dictionary learning methods

Here we extend the comparative experiments considering R-SVD (integrating OMP) versus the iterative alternating scheme method ILS-DLA, and the on-line dictionary learning method OSDL by Sulam et al. [24].

In these experiments we refer to random training sets of size *L* = 5000, 10000, dictionaries of size 64 × 128 (where the atom size is a perfect square as required in OSDL), various levels of additive noise (10, 30, 50 dB and the case of no noise), and sparsity set to 10% of the atom size. The methods are run for 200 dictionary update iterations and the obtained results are averaged over 100 trials. In Table 2 we report the average gaps between the *E*_SNR_ of R-SVD and each of ILS-DLA and OSDL, respectively. We can notice that R-SVD systematically demonstrates better performances than ILS-DLA and OSDL. More specifically, ILS-DLA shows a regular behavior, with a considerable rise of the gaps with the increase of additive noise’s SNR. Concerning OSDL, we have even more significant gaps in favor of R-SVD, but with a less regular distribution, which is not easy to be interpreted. This may be in part due to OSDL having been originally conceived for learning of large dimension image patches.

**Table 2:** Gap between *E*_SNR_ of R-SVD and each of the algorithms ILS-DLA [20] and OSDL by Sulam et al. [24] at various SNR levels of additive noise on dictionary *D* of size 64 × 128, and training size *L* = 5000, 10000.

*L*	SNR = 10		SNR = 30		SNR = 50		no noise	
	ILS-DLA	OSDL	ILS-DLA	OSDL	ILS-DLA	OSDL	ILS-DLA	OSDL
5000	0.25	2.09	2.72	0.22	2.13	4.00	3.92	4.80
10000	0.32	3.39	3.21	0.34	4.11	4.10	3.68	3.92

## 4 Experiments on natural data

In order to assess the applicability of the R-SVD method to various domains and tasks, we test it on ECG compression, EEG sparse representation, and image modeling. All the experiments are conducted on publicly available data, comparing the R-SVD and K-SVD performances, and adopting OMP as sparse decomposition method.

### 4.1 ECG compression

Sparsity techniques have been already applied to the compression of electrocardiogram (ECG) signals [31, 32]. To highlight the benefit of the dictionary learning approach in this task, we tested the two methods K-SVD and R-SVD recasting the compression as a problem of sparse approximation with a dictionary.

In this experiment, the compression process is broken down into three stages. The former is a preprocessing step consisting in R-peak detection of the signal, and its normalization (i.e., filtering, zero-padding and centering) so as to split it into *n*-length normalized RR-segments. The second stage focuses on the dictionary learning where either R-SVD or K-SVD are used to train a dictionary on a group of RR-segments taken from an initial transient of the signal (train chunk). The latter stage concerns the encoding via sparse reconstruction of all RR-segments belonging to a broader interval of the signal (test chunk). This step is carried out referring to the learnt dictionaries, and applying the OMP algorithm. Naturally, in order to make the coding step easier, magnitudes and positions of non-null sparse coefficients are handled separately.

The compression level achieved by each method is measured as compression rate (CR), that is the ratio between the number of bits of the original signal and that of the compressed representation. Assuming that the test chunk *y* is composed of *N* *q*-bit resolution samples forming *M* RR-segments, we have

CR=qN/kq^M+Mlog2mk

where *k* is the sparsity level, $\hat{q}$ denotes the bit resolution of the quantized coefficients, *m* is the number of the atoms in the dictionary, and log2(mk) is the binary coding length of coefficient positions. Being $\hat{y}$ the reconstructed version of *y*, the overall reconstruction quality measure is here specialized for ECG signal as ${\text{E}}_{\text{SNR}}=20\log_{10}\frac{{∥y∥}_{2}}{∥\hat{y}{-y∥}_{2}}$ dB (this technical change is due to the necessity of measuring the quality of signals formed by variable length segments).

**Fig 5:**
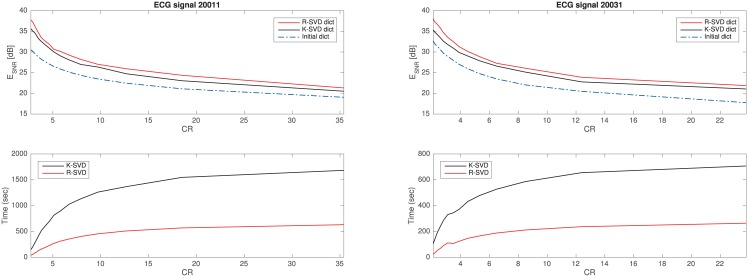
Experiments on the ECG recordings s20011 and s20031 taken from the Long-Term ST Database. (Upper plots) E_SNR_ vs CR achieved by the sparsity-based OMP compressor on dictionary learnt by R-SVD, K-SVD or on a random untrained dictionary. (Lower plots) Computational time spent by the two techniques in the learning stage.

We conducted the experiments on two ECG records taken from the Long-Term ST Database in PhysioNet [33]. They are 24 hours long recordings taken from different subjects with the aid of Holter portable devices, all sampled at *f*_*s*_ = 250 Hz and *q* = 12-bit resolution. Performances are reported in Fig 5. Upper plots show the E_SNR_ vs CR obtained from OMP, referring to dictionaries learnt by R-SVD or K-SVD, and to an untrained dictionary (i.e. randomly picking *m* RR-segments from the train chunk). Lower plots report the computational time spent by the two techniques in the learning stage. To make the experiment realistic, we set *n* = *f*_*s*_, *m* = 5*n* and $\hat{q}=q$, the dictionary training is carried out on *L* = 5000 RR-segments (about 120 minutes), while the test chunk is composed of *M* = 15000 RR-segments (about 4 hours). In order to analyze the methods at several rates, we varied *k* in the interval 5 ÷ 80 with step size 1.

Such experiments, besides confirming that trained dictionaries behave better than untrained one, prove the effectiveness of the R-SVD training method that outperforms K-SVD both in training ability and learning time.

### 4.2 EEG sparse representation

Electroencephalogram signals (EEG) are exploited in several Brain-Computer interfaces (BCI) mainly due to their high temporal resolution. An element of difficulty when dealing with this kind of signal is its non-stationary temporal behavior, an unwanted property that has negative consequences on common tasks such as classification. Several adaptive techniques and updating rules have been proposed to produce compelling dictionaries [34, 35]. Here we touch upon how dictionary learning can help in tackling these undesired aspects, allowing to produce compact and faithful signal representations.

Operatively, we split the task into three stages. The former aims at producing a pool *P* of *n*-length normalized EEG chunks corresponding to a given motor imagery (e.g. left hand, right foot). The second stage concerns the dictionary learning in the strict sense: starting from a random dictionary *D*_init_ of dimensions *n* × 2*n*, both the R-SVD and K-SVD methods are adopted to specialize *D*_init_ on the basis of a pool of EEG chunks randomly taken from *P*. Finally, the quality of the learnt dictionaries, let’s say *D*_K-SVD_, *D*_R-SVD_, is evaluated applying the OMP algorithm on a distinct pool of EEG chunks also taken from *P*.

We conducted the experiments referring to the dataset IVa from BCI competition III [36] (http://www.bbci.de/competition/iii/desc_IVa.html): EEG portions of signal corresponding to one motor imagery (either right foot or right hand) are extracted according to the given signal labelling, normalized to zero-mean signals and set into the pool *P*. The learning phase is carried out by setting the atom dimensions *n* = 150 or 300, the number of iterations *T* = 150, and expressing the sparsity *k* as various percentages of *n*. The cardinality of training set and test set is *L* = 2*n* and *M* = 4*n* respectively.

**Fig 6:**
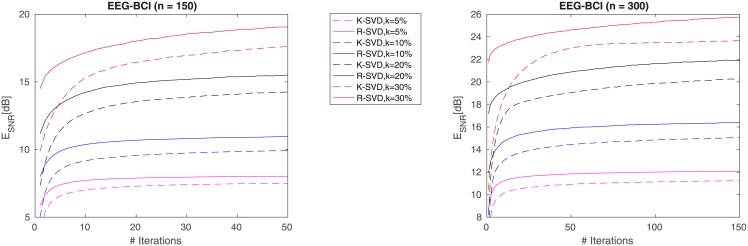
Average reconstruction error E_SNR_ for EEG signal chunks of length *n* = 150 and *n* = 300 using dictionary learnt by K-SVD (dashed lines) and R-SVD (solid lines) of dimensions *n* × 2*n*. Averages are calculated over 50 trials and plotted versus update iteration count. Considered sparsity levels: *k* = 5%, 10%, 20%, 30% of *n*.

In Fig 6 we plot the E_SNR_ trends of the learning phase on the training sets. We can observe that, R-SVD has a markedly fast gain, showing a significant gap with respect to K-SVD since the very first iterations. In Table 3 we report the reconstruction quality in terms of E_SNR_ attained in the test phase using the dictionaries *D*_K-SVD_ and *D*_R-SVD_ for sparse coding (using OMP). All results in the two phases are averaged on 50 trials. It is evident that the R-SVD method systematically exceeds K-SVD on both EEG chunk sizes. Moreover, the reconstruction quality increases with *k*, as well as the gap between the performances of K-SVD and R-SVD.

**Table 3:** Average E_SNR_ obtained on the EEG test sets. *n*: chunk dimension. %*n*: sparsity expressed as a percentage of *n*. *k*: sparsity level. *D*_K-SVD_: E_SNR_ obtained referring to the dictionary learnt by K-SVD. *D*_R-SVD_: E_SNR_ obtained referring to the dictionary learnt by R-SVD.

Reconstruction quality E_SNR_				
*n*	%*n*	*k*	*D*_K-SVD_	*D*_R-SVD_
150	0.05	8	4.78	5.21
150	0.1	15	6.55	7.19
150	0.2	30	10.08	10.84
150	0.3	45	14.05	14.78
300	0.05	15	7.73	8.47
300	0.1	30	10.92	11.88
300	0.2	60	16.73	17.82
300	0.3	90	21.38	22.64

### 4.3 Image modeling

Sparse representation of images via learnt overcomplete dictionaries is a well-known topic in the fields of image processing and computer vision [37]. In particular, in problems such as image denoising or compression [2, 38], we are interested in constructing efficient representations of patches (i.e. small portions of images) as a combination of as few as possible typical patterns (atoms) learnt from the data themselves. Naturally, the referred dictionary is crucial for the effectiveness of the method. Here, we compare performances achieved referring to the patch dictionaries learnt by either the well-known K-SVD or the R-SVD methods.

Given a pool of image patches *P*, a pre-processing is first applied aiming at both removing the patch mean intensity and reshaping all the patches to vectors in $R^{n}$.

The learning phase is carried out by setting the number of iterations to 50, and varying the sparsity *k* in 5 ÷ 30. The initial dictionaries for the two algorithms have size *n* × 1.5*n*, while training and test sets have size *L* = 2*n* and *M* = 4*n* respectively; they are all made up of randomly selected patches from *P*.

**Fig 7:**
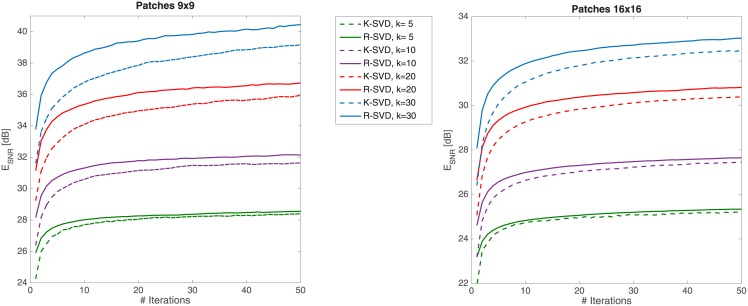
Average reconstruction error E_SNR_ for patches 9 × 9 and 16 × 16 using dictionary learnt by K-SVD (dashed lines) and R-SVD (solid lines). Averages are calculated over 50 trials and plotted versus update iteration count. Considered sparsity levels: *k* = 5, 10, 20, 30.

Experimentally, we considered patches of size 9 × 9 (*n* = 81) and 16 × 16 (*n* = 256). In both cases we randomly extracted 100,000 patches from 500 images of Caltech 101 [39] and Berkeley segmentation image database [40]. In Fig 7 we plot the E_SNR_ trends of the learning phase on the training sets. We can observe that, since the first iterations, R-SVD behaves better than K-SVD, maintaining a positive gap at convergence, especially in the cases of lower sparsity. In Table 4 we report the reconstruction quality in terms of E_SNR_ attained in the test phase using the dictionaries learnt by K-SVD and R-SVD, and then applying OMP. All results in the two phases are averaged on 50 trials. As we can observe, the R-SVD method systematically exceeds K-SVD referring to both patch sizes and to several sparsity degrees. These results further confirm the effectiveness and generality of the R-SVD method.

**Table 4:** Average E_SNR_ obtained on the image test sets. *n*: linear patch dimension. *k*: sparsity level. Last three columns are E_SNR_ achieved with initial untrained dictionary (*D*_init_), dictionary learnt by the K-SVD method (*D*_K-SVD_) and dictionary learnt by the R-SVD method (*D*_R-SVD_).

Reconstruction quality E_SNR_				
*n*	*k*	*D*_init_	*D*_K-SVD_	*D*_R-SVD_
81	5	16.76	19.74	20.16
81	10	18.34	21.94	22.22
81	20	20.78	24.73	25.12
81	30	22.88	27.38	27.65
256	5	14.84	17.40	17.74
256	10	15.91	18.96	19.27
256	20	17.49	20.96	21.21
256	30	18.45	22.02	22.34

## 5 Conclusions

In this paper we have proposed a new technique, namely R-SVD, of dictionary learning for sparse coding. It preserves the well established iterative alternating scheme adopted for example in the K-SVD algorithm: one step is for the coding of sparse coefficients, and the other one is for the dictionary optimization promoting sparsity. The main novelty of R-SVD concerns how it tackles the dictionary optimization step: instead of choosing single best atoms via SVD, it transforms groups of atoms through the best rotations found in the spirit of the Orthogonal Procrustes analysis, so as to minimize the representation error.

Extensive experiments have been conducted on both synthetic and natural data. In the former case, we investigated the behavior of R-SVD varying the atom group size and the sparse decomposition method, and we set up extensive simulations to assess the robustness and feasibility of the method, also in comparison with alternative dictionary learning algorithms. In the latter case, we considered the signal and image processing domain, showing good performances on the tasks of ECG compression, EEG sparse coding, and image modeling.

Some open issues remain to be studied. The main one is how to tackle problem (2), i.e. find the best atom group adopting more general transformations other than rotations with the Procrustes shape analysis approach. Another question concerns the resolution of problem (3) possibly through approximation techniques guaranteeing a better computational efficiency.

## Supporting Information

S1 File ECG dataset. (ZIP)

S2 File EEG dataset (chunks 150). (ZIP)

S3 File EEG dataset (chunks 300). (ZIP)

S4 File Image patches dataset. (ZIP)
